# A renewable glucose-derived methacrylate monomer for photopolymerization: synthesis, copolymerization with MMA, and structure–property relationships

**DOI:** 10.1039/d6ra01987k

**Published:** 2026-04-24

**Authors:** Rabia Nur Un, Fehmi Saltan, Gokhan Kok

**Affiliations:** a Ege University, Department of Chemistry, Faculty of Science İzmir Turkey rabia.nur.un@ege.edu.tr; b Cankiri Karatekin University, Department of Chemistry, Faculty of Science Çankırı Turkey

## Abstract

The development of sustainable polymers from renewable resources has attracted growing attention as an alternative to petroleum-based monomers. In this study, a renewable methacrylate monomer derived from protected d-glucose (MA-IPT-GF) was synthesized and copolymerized with methyl methacrylate (MMA) *via* UV-induced radical photopolymerization using benzophenone as the photoinitiator. The monomer structure was confirmed by FT-IR and NMR spectroscopy, verifying the successful introduction of the methacrylate functionality into the glucofuranose framework. Photopolymerization of MA-IPT-GF with MMA produced GF–MMA copolymers, which were characterized in terms of their structural, thermal, and morphological properties. Spectroscopic analyses revealed the disappearance of the methacrylate vinyl signals after polymerization, confirming effective copolymer formation. Thermal analysis by TG/DTG and DSC demonstrated that incorporation of the glucofuranose-derived monomer slightly reduced the onset thermal stability compared with PMMA and introduced a multistep degradation profile associated with cleavage of protecting groups and subsequent backbone decomposition. A decrease in glass transition temperature was observed, which was attributed to the presence of bulky carbohydrate side groups that disrupt efficient chain packing and increase free volume within the polymer matrix. SEM analysis revealed heterogeneous surface morphology with dispersed domains within a PMMA-rich matrix, while EDX analysis confirmed the presence of chlorine-containing protecting groups in the copolymer structure. XRD results further indicated that the copolymer maintains a predominantly amorphous structure with increased structural disorder due to the incorporation of the sugar-based monomer. Overall, the results demonstrate that glucose-derived methacrylate monomers can be incorporated into MMA-based copolymers through photopolymerization, providing renewable photocurable polymer systems.

## Abstract 2

A glucose-derived methacrylate (MA-IPT-GF) was synthesized and photopolymerized with MMA under UV irradiation using benzophenone as a photoinitiator.

## Graphical abstract

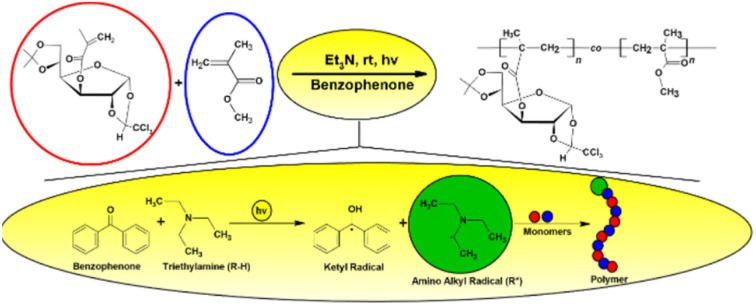

## Introduction

The increasing global demand for polymeric materials, together with growing environmental awareness, has intensified the search for more sustainable alternatives to conventional petrochemical-based monomers. Methacrylate and acrylate derivatives, such as methyl methacrylate (MMA), are widely utilized in various polymer systems due to their favorable processability and mechanical properties, and they are commonly employed in applications including coatings, adhesives, dental materials, and additive manufacturing resins. In the present study, methacrylate functionality was selected, rather than acrylate, to enable more controlled polymerization behavior and improved compatibility with MMA-based systems, particularly in the presence of bulky carbohydrate-derived structures.^1,2^ However, the production of these monomers relies heavily on fossil resources and is associated with greenhouse gas emissions and the release of volatile organic compounds (VOCs).^3^ Consequently, the development of bio-based methacrylate monomers derived from renewable feedstocks has attracted significant attention as a strategy to reduce the environmental footprint of polymer production while maintaining desirable material properties.^4^

Carbohydrates represent particularly attractive renewable platforms for monomer synthesis owing to their natural abundance, stereochemical diversity, and the presence of multiple hydroxyl groups that can be chemically functionalized.^5,6^ Various sugar-derived structures, including glucose-, mannose-, and galactose-based molecules, have been incorporated into polymer systems through esterification, etherification, or urethane linkages.^7^ The rigid and chiral nature of carbohydrate frameworks can influence polymer properties in several ways, such as increasing glass transition temperatures, enhancing intermolecular hydrogen bonding, and potentially improving biodegradability compared with petroleum-derived counterparts.^8^ For example, Molina Pinilla *et al.* reported that stereochemical differences in carbohydrate-based monomers significantly affected the thermal behavior of the resulting polymers.^9^

Several strategies have been developed to introduce methacrylate functionalities onto sugar molecules. Direct acylation using methacrylic anhydride, transesterification with glycidyl methacrylate, and protection–deprotection approaches enabling regioselective modification are among the most commonly applied methods.^10,11^ Sugar-based methacrylate monomers have been investigated for applications such as hydrogel formation,^12^ adhesive systems,^13^ and photocurable materials.^14^ Similarly, sorbitol-derived methacrylates have been copolymerized with MMA to produce polymer networks with tunable mechanical properties and enhanced hydrophilicity.^15^ Despite these advances, methacrylated glucose derivatives remain comparatively less explored within the broader field of renewable methacrylate monomers.

Most glucose-containing polymer systems reported in the literature have been investigated primarily for biomedical and glycopolymer applications due to their ability to interact with lectins and biological recognition systems.^16,17^ In this context, glucose-based polymers have often been synthesized using controlled polymerization techniques such as ring-opening polymerization (ROP)^18^ or reversible addition–fragmentation chain transfer (RAFT) polymerization.^19^ In contrast, relatively few studies have focused on the direct synthesis of methacrylated glucose monomers followed by their application in free-radical photopolymerization systems.^20^ Furthermore, many earlier studies emphasize aqueous or enzymatic polymerization approaches intended for biological applications,^21,22^ rather than photocurable formulations relevant to coatings, structural materials, or additive manufacturing technologies.

Photopolymerization is widely recognized as an efficient and environmentally favorable curing technique due to its rapid reaction rates,^23^ low energy consumption, spatial control over polymerization,^24^ and suitability for the fabrication of thin films and complex three-dimensional structures.^25^ Benzophenone is a commonly used Type II photoinitiator that generates radicals through hydrogen abstraction from suitable donors under UV irradiation, making it particularly compatible with methacrylate-based systems.^26,27^ Copolymerization of renewable monomers with MMA provides a versatile route to tailor polymer rigidity, flexibility, and thermal properties while preserving compatibility with established industrial formulations.^28–30^ Previous studies involving glucose-based methacrylate monomers copolymerized with MMA have reported changes in glass transition temperature and thermal degradation behavior depending on sugar content.^31^ suggesting that carbohydrate-derived methacrylates may provide unique structure–property relationships in methacrylate polymers. In addition, photopolymerization is particularly attractive for incorporating renewable monomers into polymer networks because it allows rapid curing under mild conditions while minimizing thermal degradation of sensitive bio-based structures. This feature makes UV-curable systems especially suitable for carbohydrate-derived monomers, whose functional groups and stereochemistry can strongly influence the resulting polymer architecture.

Despite these promising developments, systematic investigations addressing the photopolymerization behavior of glucose-derived methacrylate monomers within MMA-based copolymer systems remain scarce. In particular, the influence of protected carbohydrate structures on polymerization efficiency, copolymer architecture, and the resulting thermal and morphological properties has not yet been comprehensively explored. This lack of detailed structure–property correlation limits the rational design of renewable photocurable methacrylate systems. The study also provides comparative insights relative to other sugar-based methacrylates reported in the literature, contributing to the understanding of structure–property relationships in such systems and suggesting potential applicability in areas such as coatings,^32^ additive manufacturing,^33^ and biomedical materials.^34^

In this study, bulk photopolymerization was used as a model system to evaluate the intrinsic behavior of the monomer under UV irradiation, prior to application-oriented formulation studies. A renewable new methacrylate monomer derived from protected d-glucose was synthesized and subsequently copolymerized with MMA under UV-induced radical polymerization conditions using benzophenone as the photoinitiator. Structural characterization of the glucose-based monomer was carried out by NMR and FTIR spectroscopy to confirm successful methacrylation. The resulting monomer was then photopolymerized with MMA to obtain GF–MMA copolymers, and the resulting materials were investigated in terms of their structural, thermal, and morphological properties. To the best of our knowledge, this work represents one of the few studies investigating the UV-induced copolymerization of a protected glucose-derived methacrylate with MMA and evaluating its structure–property relationships within a photocurable polymer system.

Trichloroethylidene groups are more resistant to acidic hydrolysis than isopropylidene groups due to the electron-withdrawing effect of the chlorine atoms. While isopropylidene groups begin to hydrolyze at around pH 6, trichloroethylidene groups require more acidic conditions, with hydrolysis typically occurring at pH 4–5. This difference enables a more controlled increase in degradation lifetime, allowing biodegradable materials to be tailored to meet diverse application needs.

## Experimental section

### Materials

d-(+)-Glucose (Sigma-Aldrich, synthetic, ≥99%), chloral hydrate (Sigma-Aldrich, ≥99%), sulfuric acid (Sigma-Aldrich, ACS reagent, 95.0–98.0%), chloroform (Sigma-Aldrich, contains ethanol as stabilizer, ACS reagent, ≥99.8%), methanol (Sigma-Aldrich, ≥99.85% (GC), for synthesis), ethyl acetate (Sigma-Aldrich, ACS reagent, ≥99.5%), hexane (Sigma-Aldrich, ≥99% (GC), ACS reagent), toluene (Sigma-Aldrich, ACS reagent, ≥99.5%) dichloromethane (DCM) (Sigma-Aldrich, anhydrous, ≥99.8%, contains 40–150 ppm amylene as stabilizer), 2,2-dimethoxypropane (2,2-DMP) (Sigma-Aldrich, reagent grade, 98%, liquid, suitable for analytical testing), *p*-toluenesulfonic acid monohydrate (*p*-TSA) (Sigma-Aldrich, ACS reagent grade, ≥98.5%, solid), *N*,*N*-dimethylformamide (DMF) (Sigma-Aldrich, ACS reagent, ≥99.8%), pyridine (Sigma-Aldrich, anhydrous, 99.8%), methacrylic anhydride (Sigma-Aldrich, contains 2000 ppm topanol A as inhibitor, ≥98%), silica gel inorganic sorbent (Merck, high-purity grade (7734), 70–230 mesh), TLC plates, silica gel 60 F_254_ (Merck, plate *L* × *W* 2.5 cm × 7.5 cm, glass support).

UV-A lamp: Osram Ultra Vitalux 300 W-e27; rated voltage 300 W, rated voltage 230 V, lamp voltage 230 V, structure voltage 230 V; 400 nm (UV-A) 13.6 W, 315 nm (UV-B) 3.0 W.

### Instrumentation

NMR spectra were taken with a Varian ASV 400 MHz spectrometer at room temperature using tetramethylsilane as a standard in CDCl_3_, and chemical shifts are reported in ppm. FTIR spectra were recorded using an ATR instrument and a Mattson 1000 spectrometer. Melting points were measured with a Gallenkamp instrument. Molecular weights were determined by gel permeation chromatography (GPC) instrument equipped with a Waters Styragel column (HR series 2, 3, 5E) with THF as the eluent at a flow rate of 0.3 mL min^−1^ and a Waters 410 differential refractometer detector. TG measurements of polymer samples were obtained on PerkinElmer Diamond TA/TGA from 25 to 600 °C at heating rate 10 °C min^−1^, under constant flow rate of 100 mL min^−1^ of nitrogen atmosphere. The sample weights were taken in the range of 6–10 mg. Fourier Transform Infrared (FT-IR) spectra were recorded on a PerkinElmer FT-IR Spectrum One-B spectrometer (USA). X-Ray Diffraction (XRD) analysis was performed with a Thermo Scientific ARL K-Alpha X-ray source: Cu-Kα, normal scan speed: (0.1° 2*θ* per s), Cu radiation 1.5406 Å (0.15406 nm). All scanning electron microscopy (SEM) images were obtained using a field-emission scanning electron microscope (Thermo Scientific Apreo S SEM) under a high vacuum at a voltage of 15.0 kV and a working distance of 6.0 mm. The related samples were coated with AuPd alloy for SEM and EDX analysis by sputter coating in a 100 s diffuse technique. Images of the coated samples were obtained using an in-column secondary electron detector in the reference Polaroid 545 at an acceleration voltage of 15 kV under a high vacuum. Energy-Dispersive X-ray (EDX) analyses were performed using a 129 keV electron detector in the visible region. Images and EDX analyses were obtained with a 30 µm aperture, scanning speed of 5.4 s for a 1024 × 768 pixel image.

### Synthesis of sugar based monomer

#### Synthesis of β-chloralose (β-Cl)

A mixture of d-glucose (10 g, 50.5 mmol) and freshly distilled anhydrous chloral (6 equiv., 67 mL) was treated with one drop of sulfuric acid under an inert atmosphere and refluxed for 3 h. Reaction progress was monitored by TLC on silica gel (SiO_2_), confirming complete consumption of the starting material (*R*_f_ = 0.61, CHCl_3_/MeOH/H_2_O 61 : 32 : 7) and the formation of two distinct products (*R*_f_ = 0.18 for β-chloralose and 0.23 for α-chloralose, toluene/MeOH 8 : 2). Excess chloral was removed under reduced pressure using a rotary evaporator. The resulting dark viscous residue was dissolved in dichloromethane and filtered through Celite. The filtrate was concentrated and mixture of α- and β-chloraloses were purified by acetylation (Ac_2_O, pyridine), chromatographic separation (hexane/ethyl acetate 7 : 3) and deacetylation (MeONa, MeOH) sequence to afford β-Cl (3.28 g). Mp 234–236 °C (lit. 234–238 °C,^35^). Yield: 21%.

This method was not use any solvents. Moreover excess chloral can be easily recovered and reused *via* distillation or rotary evaporation.

#### Synthesis of 5,6-*O*-isopropylidene-1,2-*O*-(*S*)-trichloroethylidene-α-d-glucofuranose (IPT-GF)

β-Cl (3.0 g, 9.72 mmol) was dissolved in *N*,*N*-dimethylformamide (DMF, 10 mL), followed by the addition of 2,2-dimethoxypropane (2,2-DMP, 3 mL, 24.48 mmol) and *p*-toluenesulfonic acid (PTSA, 15 mg). The reaction mixture was stirred at room temperature for 7 h. After completion, the mixture was neutralized with 5% aqueous sodium bicarbonate (NaHCO_3_), and the solvent was removed under reduced pressure using a rotary evaporator. The residue was crystallized from methanol at 0 °C to afford compound 6, identified as IPT-MF 2.7 g. TLC (SiO_2_, Tol/MeOH 8 : 2): *R*_f_ = 0.41. Mp 187–189 °C (lit. 188–189 °C).^36^ Yield: 80%.

#### 5,6-*O*-Isopropylidene-3-*O*-methacroyl-1,2-*O*-(*S*)-trichloroethylidene-α-d-glucofuranse (C_15_H_19_Cl_3_O_7_) (MA-IPT-GF)

IPT-GF (1.0 g, 2.9 mmol) was dissolved in dry pyridine (6.0 mL), followed by the addition of methacrylic anhydride (1.0 mL). The reaction mixture was stirred at room temperature for 24 h. Reaction progress was monitored by TLC on silica gel using toluene/MeOH (8 : 2) as the eluent, confirming complete consumption of the starting material. The solvent was removed under reduced pressure using a rotary evaporator, and the residue was extracted with dichloromethane (CH_2_Cl_2_, 30 mL). The organic layer was dried over anhydrous sodium sulfate (Na_2_SO_4_), filtered, and concentrated.^37^ The crude product was purified by silica gel column chromatography using hexane/ethyl acetate (8 : 2) as the eluent to afford MA-IPT-GF. Yield: 68%. Mp 107.8–108.5 °C. The obtained monomer was characterized by spectroscopic techniques including ^1^H-NMR, ^13^C-NMR, and FT-IR.

##### FTIR (ATR, cm^−1^)

2936–2889 (C–H), 1728 (C=O), 1641 (C=C), 1140 (C–O–C), 809 (CH–Cl).

##### ^1^H NMR (CDCl_3_)

*δ* 6.21 (d, 1H, *J* = 4 Hz, H-1), 6.12 (bs, 1H, CH_3_–C=CH_2_), 5.66 (s, 1H, HCCCl_3_), 5.64 (d, 1H, *J*: 1.6 Hz, CH_3_–C=CH_2_), 5.39 (d, 1H, H-2), 4.90 (d, 1H, *J* = 4 Hz, H-3), 4,24 (dd, 1H, H-5), 4.19 (dd, 1H, *J*: 3.2. Hz, H-4), 4.09 (dd, 1H, *J* = 6.0 Hz, H-6a), 4.03 (dd, *J* = 4.8 Hz, H-6b), 1.95 (bs, 3H, CH_3_–C=CH_2_), 1.40 and 1.30 (s, 6H, C(CH_3_)_2_).

##### ^13^C-NMR (CDCl_3_)

*δ* 165.6 (C=O), 135.5 (CH_3_–C̲=CH_2_), 126.9 (CH_3_–C=C̲H_2_), 109.5 (C̲HCCl_3_), 109.4 (C̲(CH_3_)_2_), 106.4 (C-1), 99.3 (CHC̲Cl_3_), 85.3 (C-3), 80.7 (C-4), 75.6 (C-2), 72.2 (C-5), 67.0 (C-6), 27.4 and 26.4 (C(C̲H_3_)_2_), 18.2 (C̲H_3_–C=CH_2_).

### Synthesis of renewable glucofuranose-based methacrylate/methyl methacrylate copolymer (GF–MMA)

The copolymerization of methyl methacrylate (MMA) with the glucose-derived methacrylate monomer (MA-IPT-GF) was carried out under solvent-free conditions. MMA and MA-IPT-GF were weighed in an equimolar ratio (50 : 50 by weight) and transferred into a quartz photoreaction tube. Benzophenone (BP) was used as the photoinitiator at a concentration corresponding to 1 wt% of the total monomer content. Triethylamine (Et_3_N) was employed as a hydrogen donor in a catalytic amount. The mixture was purged with argon gas to remove dissolved oxygen, and the tube was further flushed with argon to ensure an inert atmosphere.

The photopolymerization was performed under UV irradiation using lamps emitting in both the UV-A (315–400 nm) and UV-B (280–315 nm) regions, with a minimum output power of 6 W. Benzophenone exhibits maximum absorption in the UV-B region, ensuring efficient radical generation under these conditions. The reaction was allowed to proceed for 18 h, which was determined to be the optimal reaction time. At shorter irradiation times (6 h and 12 h), only oligomeric products were obtained with insufficient molecular weight. Extending the reaction time to 24 h did not lead to further improvement in yield or molecular weight compared to 18 h. Initiator concentration was also optimized: 1 wt% benzophenone provided the most effective initiation, whereas higher loadings (2–3 wt%) resulted in excessively rapid polymerization and very low yields.

At the end of the reaction, a highly viscous liquid product was obtained. The crude product was diluted with tetrahydrofuran (THF) and precipitated dropwise into cold methanol. The resulting solid copolymer was collected by filtration, washed three times with 15 mL of methanol, and dried under vacuum at 40 °C for 24 h to yield the final copolymer as a white solid (% yield ≈ 40%, *M*_w_: 15 900 g mol^−1^, *M*_n_: 8600 g mol^−1^, PDI (*M*_w_/*M*_n_: 1.845)).

**Scheme 1:**
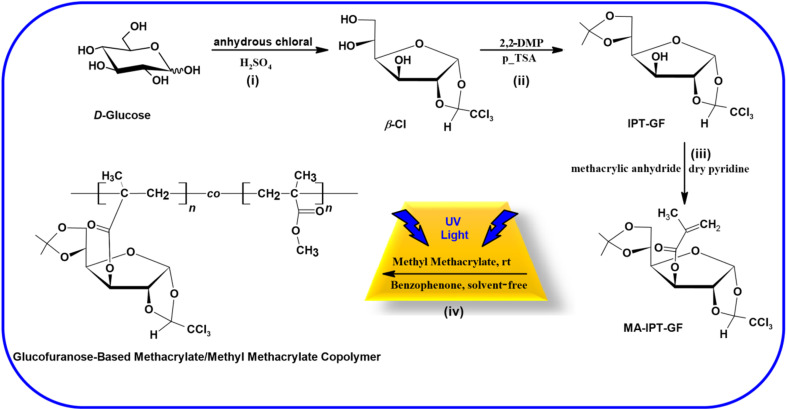
Schematic representation of the overall synthetic route: (i) synthesis of β-chloralose (β-Cl); (ii) protection to obtain IPT-GF; (iii) methacrylation to yield MA-IPT-GF; and (iv) copolymerization with MMA under UV irradiation using benzophenone as photoinitiator.

**Fig. 1:**
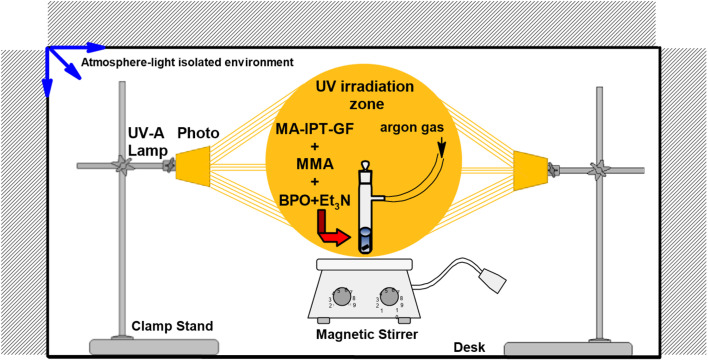
Schematic representation of the experimental setup used for the photopolymerization of MA-IPT-GF with MMA under UV irradiation in the presence of benzophenone initiator.

**Fig. 2:**
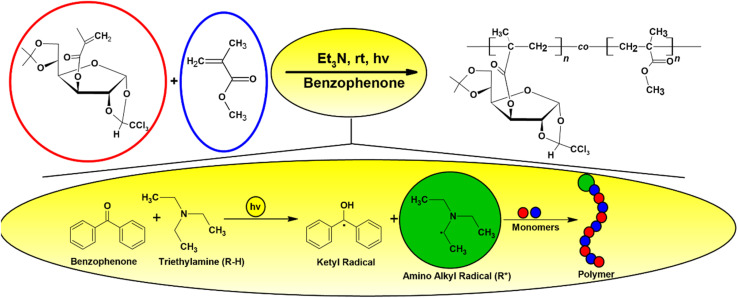
Schematic representation of the benzophenone/hydrogen donor-mediated Type II photoinitiation process.

The overall synthetic pathway, including the preparation of β-chloralose (β-Cl), protection to yield IPT-GF, methacrylation to obtain MA-IPT-GF, and subsequent copolymerization with MMA, is summarized in Scheme 1. The trichloroethylidene protecting group was employed to ensure regioselective functionalization and structural stability during polymerization, while also enabling potential post-polymerization modification. The experimental setup employed for the photopolymerization of MA-IPT-GF with MMA is illustrated in Fig. 1, while the benzophenone/hydrogen donor-mediated Type II photoinitiation mechanism responsible for radical generation under UV irradiation is schematically presented in Fig. 2.

## Results and discussions

### Spectroscopic characterization

#### FT-IR analyses

The ATR-FTIR spectrum of the methacrylated glucofuranose monomer (MA-IPT-GF) exhibits characteristic absorptions confirming successful methacrylation and the presence of the protecting groups. A strong carbonyl stretching band appears at 1728 cm^−1^, consistent with the ester C=O group of the methacrylate functionality.^37^ The residual C=C stretching vibration of the methacrylate moiety is observed at 1641 cm^−1^,^38^ while C–O–C vibrations associated with sugar ether/ester linkages appear near 1140 cm^−1^. Aliphatic C–H stretching bands are detected in the 2936–2889 cm^−1^ region. Additionally, a band at approximately 809 cm^−1^ is attributed to C–Cl vibrations originating from the trichloroethylidene protecting group, indicating that this protecting group remains intact after methacrylation.

**Fig. 3:**
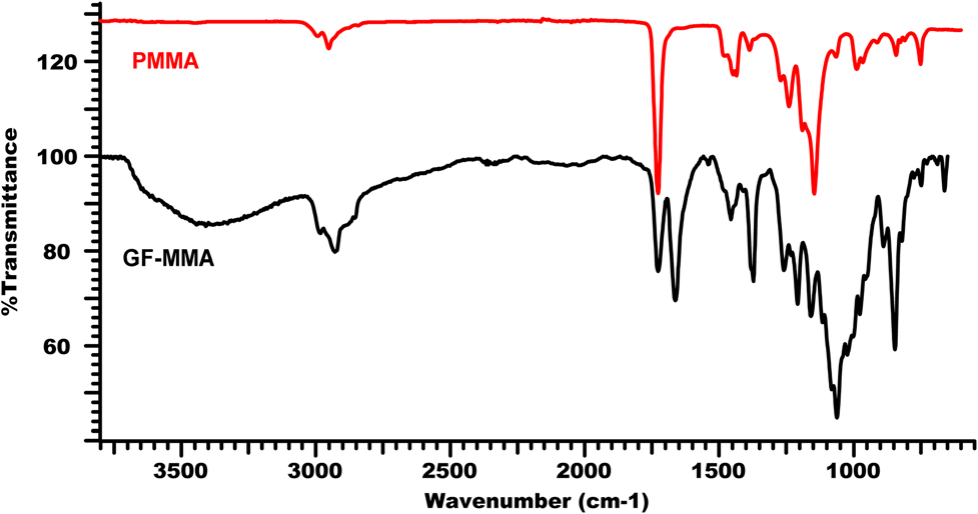
FT-IR spectra of PMMA and GF–MMA copolymer.

Comparison of the FT-IR spectra of the GF–MMA copolymer with neat PMMA, shown in Fig. 3, reveals the expected spectroscopic changes associated with polymerization. The characteristic methacrylate vinyl C=C band (∼1640 cm^−1^) present in the monomer is absent or significantly reduced in the copolymer spectrum, indicating the consumption of the double bonds during radical polymerization. The ester carbonyl band remains visible in the copolymer spectrum with little or no shift from ∼1728 cm^−1^, confirming retention of the ester functionality within the polymer backbone.^39^ Furthermore, the C–O–C region (∼1140 cm^−1^) is preserved and typically appears broader due to overlapping contributions from both PMMA ester groups and sugar ether linkages.^40^ Notably, the persistence of the ∼809 cm^−1^ band in the copolymer spectrum indicates that the trichloroethylidene protecting group (and the associated C–Cl functionalities) remains chemically intact during the photocuring process. Together, these spectral features confirm successful copolymer formation while demonstrating that the protecting groups were not cleaved during photopolymerization.

#### NMR analyses

**Fig. 4:**
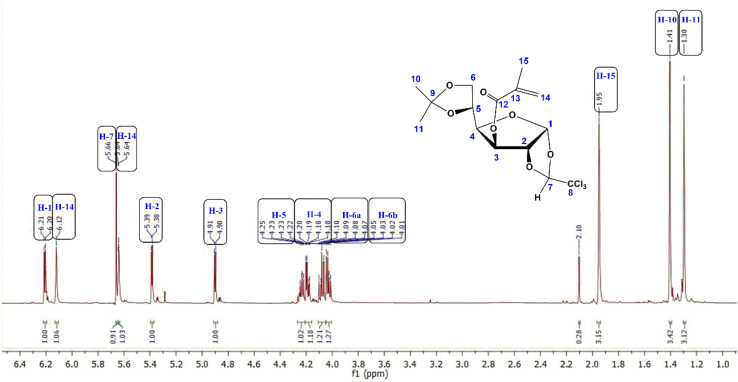
^1^H-NMR spectra of the MA-IPT-GF monomer.

The ^1^H-NMR spectrum of MA-IPT-GF (CDCl_3_) shows signals characteristic of the methacrylate substituent together with resonances corresponding to the glucofuranose core (Fig. 4). The vinylic protons of the methacrylate group appear at *δ* 6.12 (broad s, 1H) and *δ* 5.64 (d, *J* = 1.6 Hz, 1H), while the methyl group of the methacrylate vinyl (–CH_3_) resonates as a broadened singlet at *δ* 1.95 (3H).

The anomeric proton (H-1) is observed at *δ* 6.21 (d, *J* = 4 Hz, 1H), and the remaining sugar ring protons appear in the *δ* 4.90–4.03 region, as expected for a protected glucofuranose framework.^20^ These resonances confirm both the integrity of the sugar ring and the successful introduction of the methacryloyl functionality.

The ^13^C-NMR spectrum is consistent with the proposed structure. The ester carbonyl carbon appears at *δ* 165.6, while the vinyl carbons of the methacrylate group are observed at *δ* 135.5 and 126.9. Signals associated with CHCl_3_-derived centers and protected sugar carbons are detected in the ranges *δ* 109–107 and *δ* 85–67, respectively, whereas the isopropylidene methyl carbons appear at *δ* 27.4 and 26.4. These chemical shifts collectively support the presence of both the glycosidic framework and the methacryloyl substituent.

**Fig. 5:**
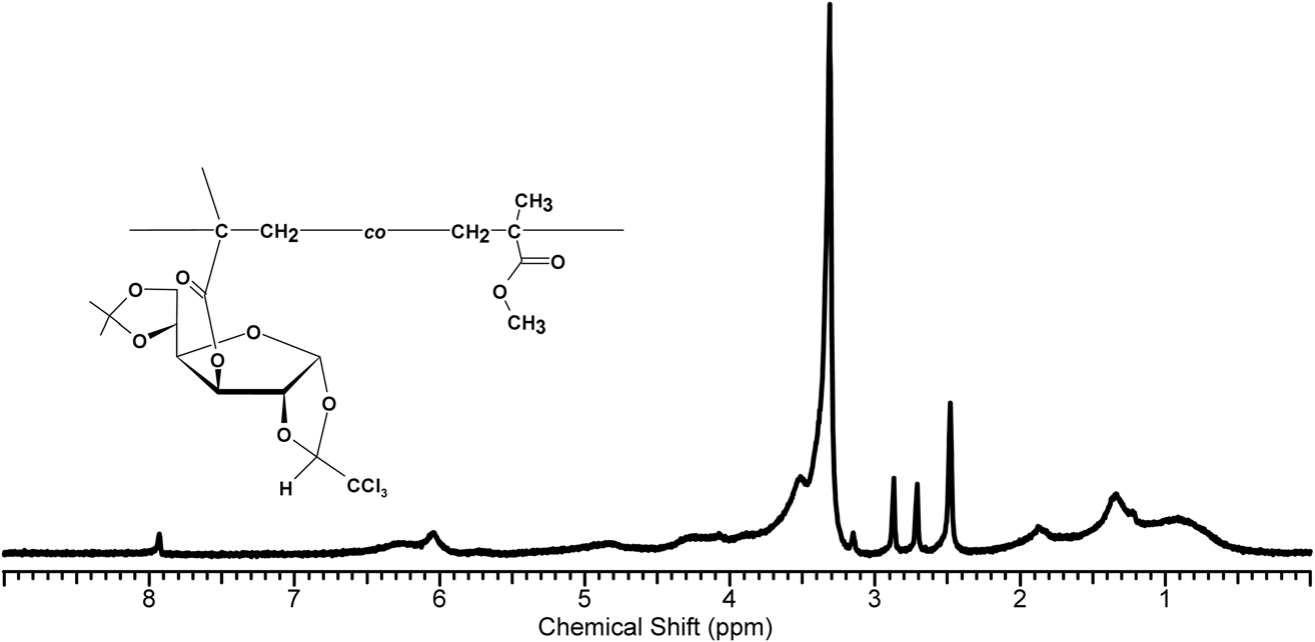
^1^H-NMR spectra of the GF–MMA copolymer.

The ^1^H-NMR spectrum of the GF–MMA copolymer as shown in Fig. 5 exhibits the characteristic features of an MMA-containing polymer together with broadened signals attributable to the incorporated glucofuranose units.^41^ Most notably, the monomeric vinylic signals of MA-IPT-GF at *δ* 6.12 and *δ* 5.64 disappear (or are reduced to baseline), indicating extensive consumption of the vinyl groups and successful radical polymerization. The MMA methoxy protons (–OCH_3_) appear near *δ* 3.6 as a singlet and serve as a convenient reference signal for copolymer composition analysis. Although precise copolymer composition determination was not the primary focus of this study, the relative integration of the methoxy signal of MMA and the anomeric proton of the glucofuranose unit suggests successful incorporation of the sugar-derived monomer into the polymer backbone. Signals originating from the sugar units, originally located between *δ* 4.0–5.0, remain detectable but become broadened and slightly shifted due to incorporation into the macromolecular environment. In particular, the anomeric resonance becomes less sharp but can still be identified, supporting the presence of glucofuranose segments within the copolymer structure. The persistence of these sugar-derived resonances, together with the disappearance of the methacrylate vinyl signals, provides clear spectroscopic evidence that the MA-IPT-GF units are covalently incorporated into the MMA-based copolymer backbone rather than remaining as unreacted monomer. Although precise copolymer composition determination was not the primary focus of this study, a semi-quantitative analysis was performed based on the relative integration of the characteristic ^1^H-NMR signals assigned to the methoxy protons of MMA (around 3.6 ppm, 3H) and the anomeric proton of the glucofuranose unit (around 6.2 ppm, 1H). Considering the broadened nature of the polymer signals, the copolymer composition was estimated to be approximately 32 mol% GF-derived units and 68 mol% MMA units. This deviation from the feed ratio (50 : 50) suggests a relatively lower incorporation efficiency of the bulky glucose-based monomer, likely due to steric hindrance and reduced reactivity during radical copolymerization.

Taken together, the FT-IR and NMR results provide clear evidence for the successful methacrylation of the protected glucofuranose monomer (MA-IPT-GF) and its subsequent incorporation into MMA-based copolymers. The disappearance of methacrylate vinyl signals in both FT-IR and ^1^H-NMR spectra, together with the persistence of the ester carbonyl band, confirms effective radical copolymerization under benzophenone-mediated UV irradiation. Furthermore, the presence of trichloroethylidene-related C–Cl signals in FT-IR and protected-sugar carbon resonances in NMR indicates that the protecting groups remain intact during the photopolymerization process, which may be advantageous for potential post-functionalization or deprotection steps.

#### GPC analysis

The relatively moderate molecular weight (*M*_w_ ≈ 15 900 g mol^−1^) and dispersity (PDI ≈ 1.845) observed for the GF–MMA copolymer can be attributed to the presence of the bulky and highly functionalized glucose-derived monomer. The steric hindrance introduced by the protected glucofuranose units likely reduces the propagation efficiency during radical polymerization, leading to limited chain growth.

In addition, the presence of bulky and polar carbohydrate moieties may increase the probability of premature termination, particularly through bimolecular radical recombination or disproportionation, due to restricted chain mobility and increased local viscosity. Such effects can hinder effective chain propagation and contribute to the formation of shorter polymer chains. Furthermore, diffusion limitations arising from the incorporation of rigid and polar side groups may reduce the mobility of growing radical species, thereby increasing the likelihood of termination events.

Compared to conventional PMMA systems, these results indicate that the incorporation of the glucose-based monomer significantly influences the polymerization kinetics and molecular architecture of the resulting copolymer, leading to lower molecular weight and broader molecular weight distribution.

The obtained molecular weight values are lower than those typically reported for conventional PMMA prepared *via* free radical polymerization, where significantly higher molecular weights are often achieved under similar conditions. This difference can be attributed to the presence of the bulky and sterically demanding glucose-derived monomer, which reduces propagation efficiency and promotes earlier termination. Similar trends have been reported in the literature for carbohydrate-based methacrylate systems, where the incorporation of sugar-derived units leads to reduced molecular weight and broader dispersity due to steric effects and increased polarity of the comonomer.^41,42^ These comparisons further support the conclusion that the glucose-based monomer plays a key role in controlling the molecular architecture of the resulting copolymer.

#### Thermal analysis

**Fig. 6:**
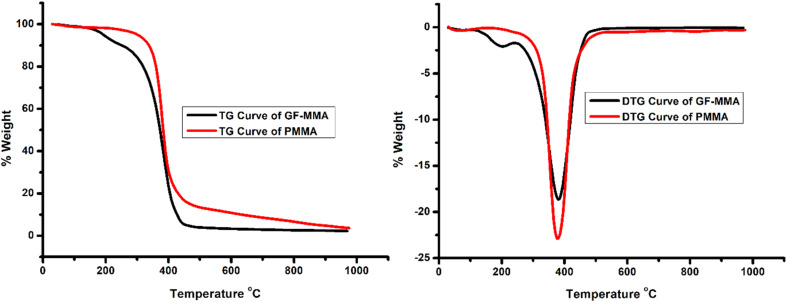
TG and DTG curves of GF–MMA and PMMA.

The thermal stability of the GF–MMA copolymer was evaluated by TG/DTG and DSC analyses, as shown in Fig. 5 and 6, respectively. The TG/DTG curves reveal a multistep decomposition profile for GF–MMA. An initial low-temperature mass loss is attributed to the cleavage of acetal and trichloroethylidene protecting groups, followed by a major high-temperature decomposition stage associated with backbone depolymerization, with DTG maxima observed at approximately 200 °C and 400 °C, respectively.^43,44^ Compared with PMMA, the GF–MMA copolymer exhibits a lower onset decomposition temperature (∼200 °C),^45^ indicating that the incorporation of glucofuranose-derived units slightly reduces thermal stability under inert conditions.

Thermogravimetric analysis further reveals a marked difference in char formation behavior between PMMA and the GF–MMA copolymer (Fig. 6). While PMMA retains approximately 20% residue at around 470 °C under a nitrogen atmosphere, the GF–MMA copolymer exhibits nearly zero char yield at the same temperature. This behavior can be attributed to the presence of sugar-derived pendant groups and protecting units (isopropylidene and trichloroethylidene), which undergo early-stage cleavage and generate volatile degradation products, thereby limiting the formation of carbonaceous residue.^43,44^ In addition, the relatively lower molecular weight of the copolymer and the possible release of halogen-containing volatile species from the trichloroethylidene group may further contribute to the reduced char yield.

In parallel, the decrease in glass transition temperature (*T*_g_) observed for the GF–MMA copolymer compared to PMMA can be explained by the incorporation of bulky glucofuranose-derived side groups, which disrupt efficient chain packing and increase the free volume within the polymer matrix. This effect enhances segmental mobility and results in a lower *T*_g_. Similar trends have been reported for carbohydrate-based methacrylate systems, where bulky and flexible pendant groups reduce intermolecular interactions and packing efficiency.^31^

**Fig. 7:**
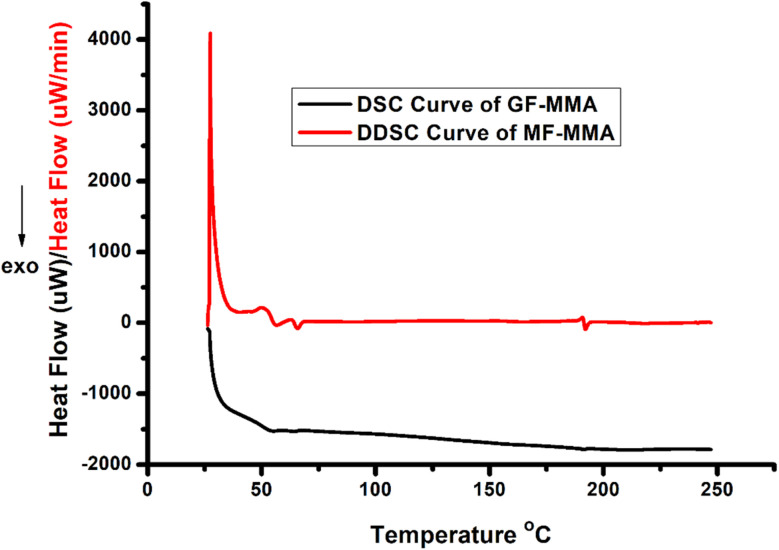
DSC and DDSC curves of GF–MMA.

DSC/DDSC measurements show a glass transition temperature (*T*_g_) of approximately 51 °C for the GF–MMA copolymer (Fig. 7). Relative to PMMA, a decrease in *T*_g_ is observed. This behavior is attributed to the incorporation of bulky sugar-based side groups that disturb efficient chain packing and increase the free volume within the polymer matrix, thereby facilitating segmental motion at lower temperatures. A weak exothermic event detected near 70 °C in the DDSC trace correlates with the low-temperature DTG feature and is consistent with thermally activated deprotection or structural rearrangement occurring prior to the main-chain degradation. In addition, DSC analysis does not reveal multiple glass transition temperatures, which would typically be expected in the case of well-defined phase separation. This observation further supports that the system should be described in terms of structural heterogeneity rather than distinct phase-separated domains.

Overall, these results indicate that the incorporation of glucofuranose-derived units modifies both the degradation pathway and thermal characteristics of the MMA-based polymer system. This behavior highlights the significant influence of carbohydrate-derived side groups on the thermal response of methacrylate polymers, demonstrating how the incorporation of renewable monomer units can alter degradation pathways and macromolecular packing within the polymer matrix.

#### SEM and EDX analyses

**Fig. 8:**
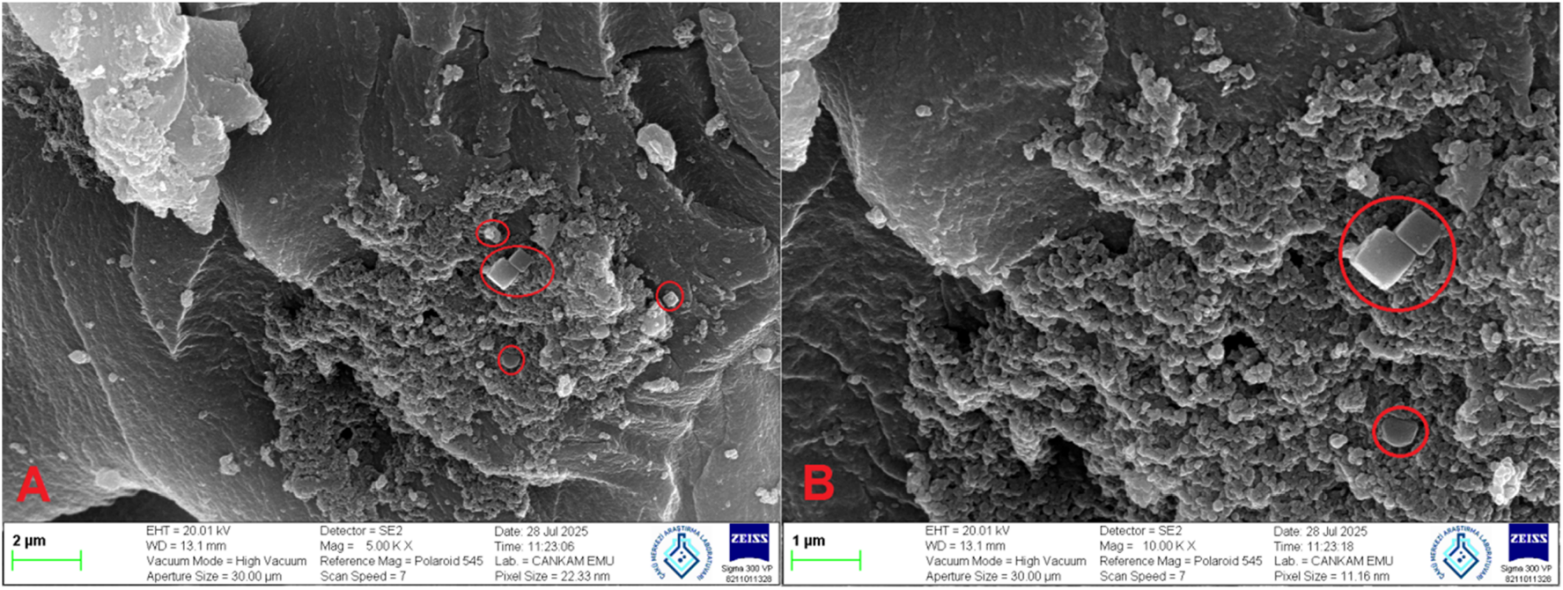
SEM images of GF–MMA (A-2 µm, B-1 µm).

As shown in Fig. 8, the SEM microstructure of the GF–MMA copolymer reveals a heterogeneous surface characterized by dispersed features within a continuous polymer matrix. These regions appear as slightly elevated or irregular areas compared with the relatively smoother PMMA-rich phase, suggesting possible limited compatibility between the glucofuranose-derived MA-IPT-GF units and the MMA backbone. The presence of sugar-based pendant groups introduces polarity differences within the polymer structure, which may promote localized aggregation through dipole–dipole interactions and preferential association of the protected carbohydrate moieties. Similar surface features have been reported for saccharide-containing methacrylate systems, where steric bulk and hydrophilic functional groups can disturb uniform chain packing and produce distinct contrasts observable by SEM.^41,42^

The observed topographical variation therefore indicates the presence of compositionally heterogeneous regions at the sub-micron scale, which may be associated with structural heterogeneity arising from the incorporation of a highly functionalized bio-based monomer into a predominantly hydrophobic PMMA framework.^42^ However, it should be noted that SEM provides only surface-level morphological information, and therefore these observations should be interpreted cautiously. More detailed analyses using techniques such as TEM, AFM, or SAXS would be required to conclusively determine the phase behavior of the system.

EDX analysis, summarized in Table 1, provides additional support for the incorporation of the MA-IPT-GF monomer into the GF–MMA copolymer. The elemental composition shows high carbon and oxygen contents, which are consistent with contributions from both the MMA backbone and the oxygen-rich sugar/acetal functionalities of the glucofuranose derivative. Most notably, a significant chlorine signal—absent in PMMA—is detected, indicating the presence of the trichloroethylidene protecting group originating from MA-IPT-GF. If this protecting group had undergone photochemical cleavage during polymerization, the chlorine signal would be expected to decrease significantly or disappear. The persistence of a strong Cl peak therefore suggests that the protecting group remains chemically intact during the UV curing process. These elemental findings are consistent with a copolymer structure in which MA-IPT-GF units are incorporated into the PMMA matrix while preserving their characteristic functional groups. The combined SEM and EDX results therefore indicate the presence of chemically distinct regions containing glucose-derived units with chlorine-bearing functionalities within the polymer structure.

**Table 1:** EDX results of GF–MMA

Element	Weight%	% atomic
C K	67.97	79.39
O K	16.51	14.47
Cl K	15.52	6.14

**Fig. 9:**
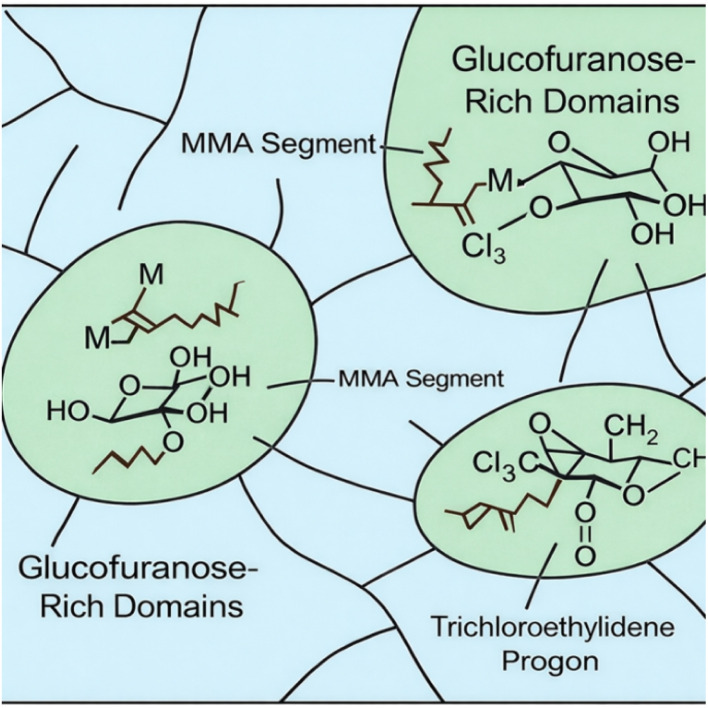
Schematic representation of the proposed heterogeneous microstructure of the GF–MMA copolymer.

Based on these observations, a schematic representation of the GF–MMA copolymer microstructure is proposed in Fig. 9. In this conceptual model, the MMA segments form the primary polymer backbone, generating a hydrophobic and glassy continuous phase, while the MA-IPT-GF units introduce bulky and highly oxygenated saccharide pendants together with trichloroethylidene protecting groups. Owing to their increased polarity and steric bulk, these glucose-derived segments may exhibit reduced compatibility with the MMA chains,^42,46^ potentially leading to the formation of localized clusters or heterogeneous domains dispersed within the PMMA-rich matrix. The schematic representation is intended as a qualitative model consistent with the SEM observations and EDX elemental distribution, reflecting the intrinsic thermodynamic and steric differences between the bio-based comonomer and the methacrylate backbone.

#### XRD analyses

**Fig. 10:**
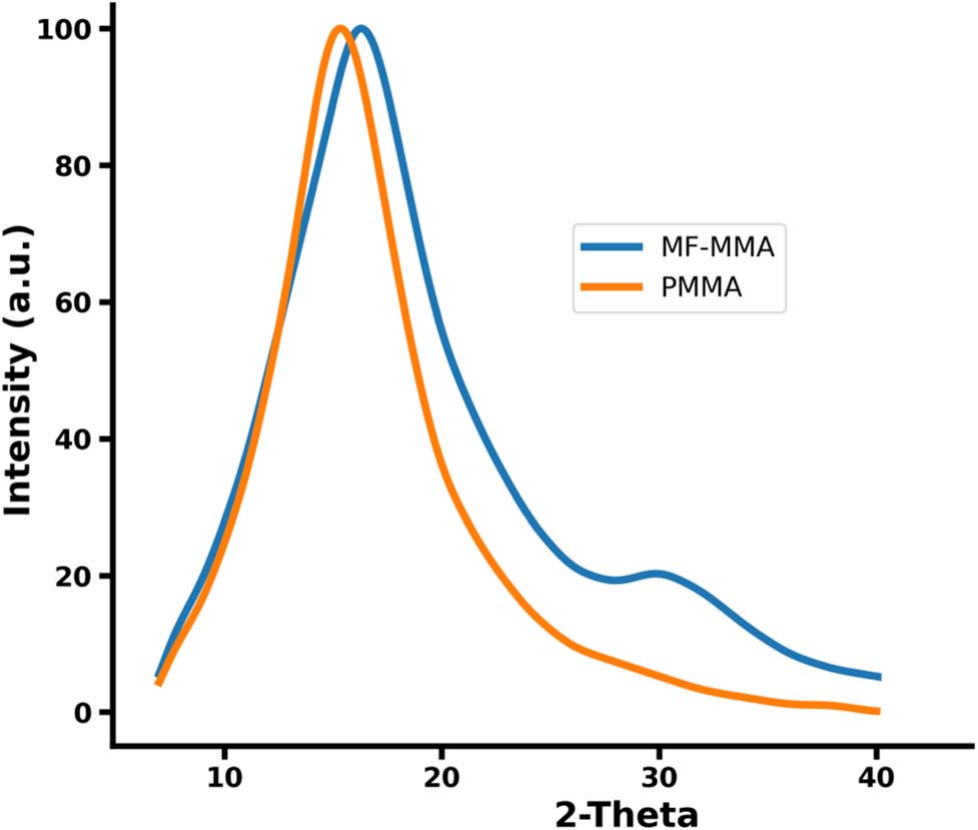
XRD patterns of the GF–MMA and PMMA.

The X-ray diffraction patterns of PMMA and the GF–MMA copolymer, shown in Fig. 10, exhibit the characteristic features of amorphous polymeric materials, with both samples displaying a broad diffraction maximum and no evidence of long-range crystalline order.^47,48^ PMMA shows a relatively narrow amorphous halo centered around 2*θ* ≈ 15°, consistent with the well-established amorphous nature of atactic polymethyl methacrylate. This broad feature is smooth and devoid of sharp Bragg reflections, confirming the absence of periodic chain packing and indicating a predominantly glassy structure.^49^ In comparison, the GF–MMA copolymer presents a similarly amorphous diffraction pattern but with noticeable differences in peak shape and position. The main diffraction maximum of the copolymer is slightly shifted toward higher 2*θ* values (∼16°) and appears broader and less defined than that of PMMA. Rather than indicating a transition to a more disordered structure, these changes suggest modifications in short-range chain packing and local molecular arrangement.

The incorporation of bulky and polar glucofuranose-derived side groups can interfere with efficient chain packing, alter intermolecular spacing, and introduce local density fluctuations within the polymer matrix. Such effects are expected to broaden the amorphous halo and slightly shift its position, as observed here. Similar behavior has been reported for glycopolymers and carbohydrate-based methacrylate systems, where sterically demanding substituents influence local packing without inducing crystallinity.

Additionally, the GF–MMA diffractogram exhibits a weak shoulder in the 28–34° region, which may be associated with the presence of multiple local packing environments rather than true crystalline domains. This feature is likely related to differences in electron density between regions enriched in sugar-derived units and the MMA-rich matrix, consistent with the heterogeneous morphology suggested by SEM observations.

Therefore, the XRD results should be interpreted as evidence of changes in short-range structural organization and local packing characteristics, rather than an increase in overall structural disorder relative to PMMA. These findings support the conclusion that the incorporation of the glucose-based monomer modifies the local molecular arrangement within an otherwise amorphous polymer matrix.

## Conclusion

In this study, a renewable methacrylate monomer derived from protected d-glucose (MA-IPT-GF) was successfully synthesized and subsequently copolymerized with methyl methacrylate (MMA) *via* UV-induced radical photopolymerization using benzophenone as a photoinitiator. Structural characterization by FT-IR and NMR spectroscopy confirmed the successful introduction of the methacrylate functionality into the glucofuranose derivative and verified its incorporation into the MMA-based copolymer network. The disappearance of methacrylate vinyl signals together with the preservation of characteristic sugar-related resonances demonstrated effective copolymer formation.

Thermal analysis revealed that the incorporation of glucofuranose-derived units slightly reduced the onset thermal stability of the copolymer relative to PMMA, while introducing distinct multistep degradation behavior associated with the cleavage of protecting groups and subsequent backbone decomposition. The observed decrease in glass transition temperature was attributed to the presence of bulky carbohydrate side groups that disturb efficient chain packing and increase the free volume within the polymer matrix.

Morphological analysis by SEM indicated a heterogeneous microstructure containing dispersed domains within a continuous PMMA-rich phase, suggesting limited compatibility between the carbohydrate-derived units and the MMA backbone. EDX analysis further confirmed the presence of chlorine-containing trichloroethylidene groups within the polymer, demonstrating that the protecting groups remained intact during photopolymerization. XRD measurements revealed that both PMMA and GF–MMA exhibit predominantly amorphous structures, while the incorporation of the sugar-based monomer increased structural disorder due to steric and polarity differences introduced by the glucofuranose side groups.

Overall, the results demonstrate that protected glucose-derived methacrylate monomers can be successfully incorporated into MMA-based polymer systems *via* photopolymerization, leading to the formation of renewable copolymers with altered structural and thermal properties. These findings offer important insight into the influence of protected carbohydrate-based methacrylate units on polymerization behavior and resulting material characteristics, thereby supporting the rational design of sustainable photocurable polymer systems. Further studies involving formulated systems (*e.g.*, coatings or photocurable resins) are required to evaluate the application performance of the developed monomer.

## Author contributions

Rabia Nur Un: writing – original draft, validation, methodology, investigation. Fehmi Saltan: writing-original draft, validation, methodology, investigation. Gokhan Kok: writing – original draft, validation, methodology, investigation.

## Conflicts of interest

There are no conflicts to declare.

## Supplementary Material

RA-016-D6RA01987K-s001

## Data Availability

The authors confirm that the data used to support the findings of this study are included within the article and are available from the corresponding author upon reasonable request. Supplementary information (SI): the GPC chromatogram of the GF-MMA copolymer and the ^13^C NMR spectra of the MA-IPT-GF monomer. See DOI: https://doi.org/10.1039/d6ra01987k.
