# Supplementary material for: A renewable glucose-derived methacrylate monomer for photopolymerization: synthesis, copolymerization with MMA, and structure–property relationships
Source: RSC Adv. 2026 Apr 24;16(24):21549–60. doi: 10.1039/d6ra01987k (PMC13107158; doi:10.1039/d6ra01987k)
Supplement: RA-016-D6RA01987K-s001 [file RA-016-D6RA01987K-s001.pdf]

Supplementary Information (SI) for RSC Advances.
This journal is © The Royal Society of Chemistry 2026

Renewable Sugar-Based Methacrylate Monomer for Photopolymer Application: Synthesis and Copolymerization with MMA

Rabia Nur ÜN[1*], Fehmi SALTAN[2], Gökhan KÖK[1]

[1]Ege University, Department of Chemistry, Faculty of Science, İzmir, Turkey

[2]Cankiri Karatekin University, Department of Chemistry, Faculty of Science, Çankırı, Turkey

rabia.un@ege.edu.tr

SUPPORTING INFORMATION

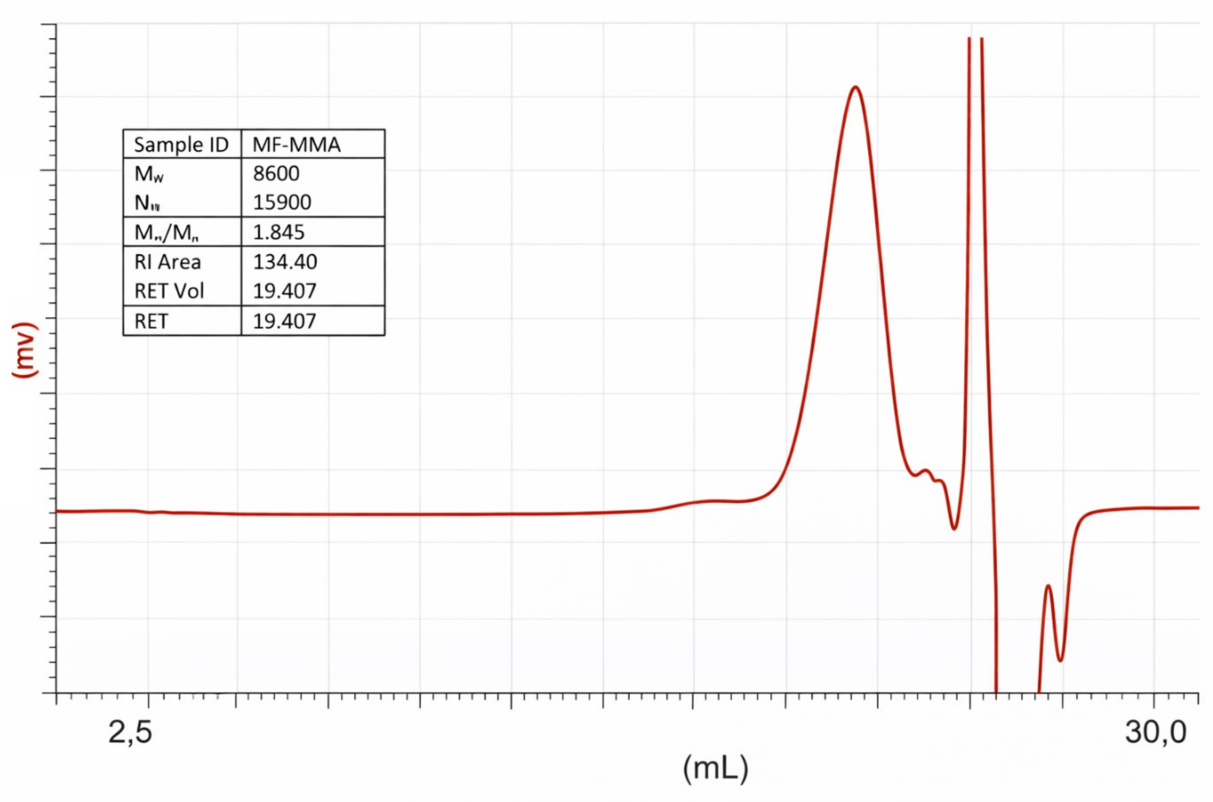

Figure S1. GPC chromatogram of the GF–MMA copolymer showing molecular weight distribution (Mw, Mn, and PDI values).

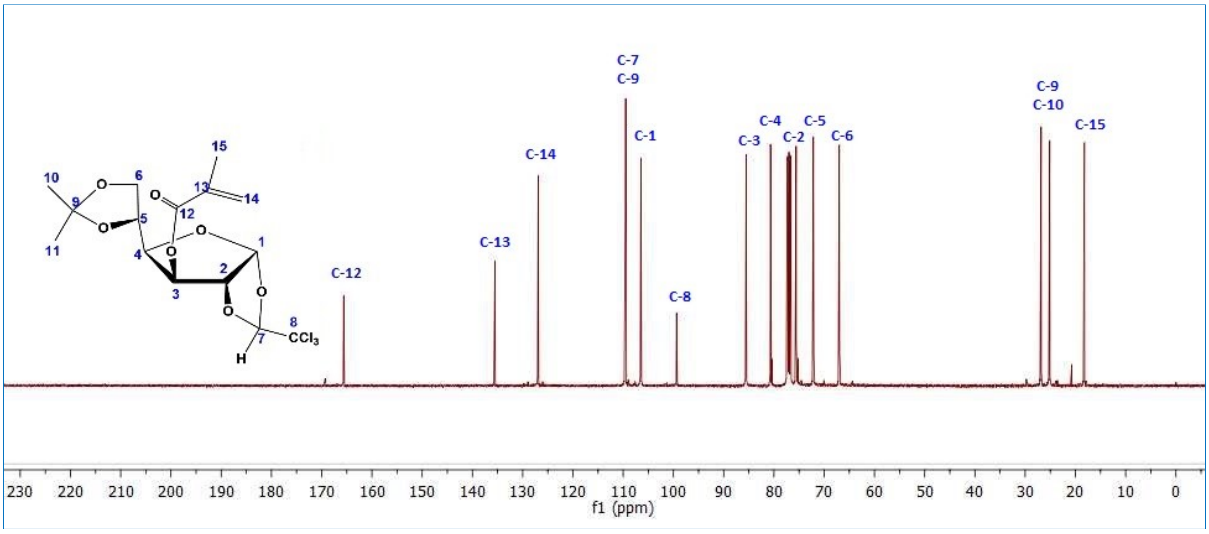

Figure S2. ^{13}C-NMR spectra of the MA-IPT-GF monomer
